# Combined ReaxFF and Ab Initio MD Simulations of Brown Coal Oxidation and Coal–Water Interactions

**DOI:** 10.3390/e24010071

**Published:** 2021-12-31

**Authors:** Shi Yu, Ruizhi Chu, Xiao Li, Guoguang Wu, Xianliang Meng

**Affiliations:** 1 Department of Chemical Engineering, China University of Mining & Technology, Xuzhou 221116, China; 5804@cumt.edu.cn (S.Y.); rzchu@cumt.edu.cn (R.C.); lixiao@cumt.edu.cn (X.L.); ggwu@cumt.edu.cn (G.W.); 2 Key Laboratory of Coal-Based CO_2_ Capture and Geological Storage, China University of Mining & Technology, Xuzhou 221116, China

**Keywords:** ReaxFF, CPMD, brown coal oxidation, ab initio molecular dynamics

## Abstract

In this manuscript, we use a combination of Car–Parrinello molecular dynamics (CPMD) and ReaxFF reactive molecular dynamics (ReaxFF-MD) simulations to study the brown coal–water interactions and coal oxidation. Our Car–Parrinello molecular dynamics simulation results reveal that hydrogen bonds dominate the water adsorption process, and oxygen-containing functional groups such as carboxyl play an important role in the interaction between brown coal and water. The discrepancy in hydrogen bonds formation between our simulation results by ab initio molecular dynamics (CPMD) and that by ReaxFF-MD indicates that the ReaxFF force field is not capable of accurately describing the diffusive behaviors of water on lignite at low temperatures. The oxidations of brown coal for both fuel rich and fuel lean conditions at various temperatures were investigated using ReaxFF-MD simulations through which the generation rates of major products were obtained. In addition, it was observed that the density decrease significantly enhances the generation of gaseous products due to the entropy gain by reducing system density. Although the ReaxFF-MD simulation of complete coal combustion process is limited to high temperatures, the combined CPMD and ReaxFF-MD simulations allow us to examine the correlation between water adsorption on brown coal and the initial stage of coal oxidation.

## 1. Introduction

Coal continues to be the second largest fuel, which supplies 27% of global primary energy needs [1]. The major consumer of coal resources is coal-fired power plants. In addition, brown coal is one of the most widely used combustion fuel sources in electric power generation, which can be attributed to its abundant reserves and low mining cost [2]. However, the high inherent moisture of low-rank coals (e.g., lignite) obstructs the large-scale utilization of low-rank coals [3], as the high water content degrades the energy quality of the coal and results in extra costs for transportation. Hence, upgrading brown coal by reducing its water content is necessary [4]. Moreover, brown coal with complex carbonaceous structures is at a high risk of spontaneous combustion, since brown coal has a large amount of oxygen-containing polar functional groups (including carboxyl, hydroxyl, and carbonyl) [2,5]. Therefore, revealing the relationship between brown coal chemical structures and its oxidation at various temperatures can throw some light on brown coal spontaneous combustion and gasification [6]. In addition, those polar functional groups also play an important role in water adsorption [2,3,7]. Thus, it is of great interest to investigate how the water adsorption is affected by the chemical structure of brown coal.

Extensive ab initio calculations (e.g., DFT) have been carried out to investigate the interactions between coal and water molecules [3,8,9]. Gao et al. [8] obtained several typical conformations of water–lignite complexes and corresponding electrostatic potential distributions using DFT calculations. In addition, they have observed that hydrogen bonding dominates the water adsorption on lignite surface, in which water molecules tend to aggregate around oxygen-containing polar functional groups. Xiao et al. [9] examined the interactions between water molecules and oxygen/nitrogen atoms in a lignite model molecule with a DFT and AIM study, and they demonstrated that water clusters play an important role in water–lignite interaction. Although such ab initio studies of brown coal–water interaction provide detailed structural information about water adsorption on lignite, it is still challenging to access the dynamical information about water transportation within brown coal under different conditions. Additionally, the information about entropy is lacking in those calculations. On the other hand, some coarser models based on percolation theory have been developed to study the coal combustion process [10] as well as coal pyrolysis [11], using the coal’s industrial analysis value. To bridge the gap between such crude models and the computationally expensive ab initio studies, some molecular dynamics simulation techniques which can be used to explore the reactive systems might be required. More importantly, since the water adsorption on lignite, water/oxygen transportation within lignite, and lignite oxidation are all correlated, reactive molecular dynamics simulations might allow us to investigate the coal desiccation, spontaneous combustion, and gasification without arbitrary assumptions. Furthermore, reactive molecular dynamics simulations have an advantage over the equilibrium modeling of coal oxidation (e.g., gasification [12]), which employs the free energy minimization method in investigating the systems far from equilibrium.

In the last two decades, the ReaxFF reactive force field developed by van Duin et al. [13,14] has wide applications in simulating high energy materials [15,16,17], hydrocarbon oxidation [18], fracture of graphene [19], and thermal fragmentation of fullerenes [20], etc. ReaxFF relates the bond order/bond energy to separations between atoms so that bonds’ formation/dissociation can take place at an appropriate distance. In addition, the parameterization of ReaxFF reactive force fields was based on the DFT calculations to determine bond energy [13]. Since the ReaxFF reactive molecular dynamics is about two orders of magnitude faster compared to quantum mechanics calculations [13,21,22], ReaxFF in combination with proper coal models [23,24] has a great potential in simulation of coal oxidation. During the last decade, ReaxFF was used to examine coal pyrolysis [25,26,27,28,29,30,31], lignin pyrolysis [32], petroleum coke pyrolysis [33], coal combustion [22,34], coal gasification [6,35], and coal tar pitch oxidation [36]. The decomposition of coal molecules to produce small fragments [34] as well as pathways of oxidation [35] have been carefully studied by ReaxFF-MD. However, most of those studies focus on high-temperature oxidation/reactions of coal molecules. Hence, the effects of interaction between water/oxygen and coal on low-temperature coal oxidation have not been fully explored by simulations at a molecular level.

Many studies on diffusion of different gases on coal using molecular dynamics simulations with conventional nonreactive force fields (e.g., COMPASS [37,38], Dreiding [39]) have been reported recently. Although the adsorption and diffusive behaviors of water, oxygen, and other gases on coal can be accurately captured through such conventional molecular dynamics simulations, the coupling between adsorption and oxidation cannot be investigated in such MD simulations, since the covalent bonds are unbreakable in those conventional force fields. A recent research on ab initio molecular dynamics simulation of lignite–water interaction has been carried out [40], which might overcome the limitations of nonreactive MD. Using the Car–Parrinello molecular dynamics simulations, Gao et al. [40] explored the adsorptions of water molecules on lignite surface and observed the replacement phenomenon of the water during a very short period of time. Moreover, their research can provide information about statistical properties of the systems which is lacking in previous DFT studies [3,8,9]. In addition, the Car–Parrinello molecular dynamics simulations results can be useful in verifying ReaxFF-MD simulations, since the training dataset for ReaxFF parametrization does not contain all possible reactions.

In this manuscript, a combination of ab initio molecular dynamics (Car–Parrinello molecular dynamics) simulations and ReaxFF reactive molecular dynamics simulations was adopted to investigate brown coal–water interaction as well as coal oxidation at various temperatures. As both DFT studies [8,9] and ab initio molecular dynamics simulations [40] demonstrated that the hydrogen bonds play a very important role in brown coal–water interactions, the hydrogen bond formation between the molecular model of brown coal and water molecule was explored by both Car–Parrinello MD and ReaxFF-MD in this work. In order to examine the brown coal oxidation under different conditions, only ReaxFF-MD simulations were performed, for the Car–Parrinello MD simulations are too computationally expensive for large systems. Although it is of great interest to study how the accumulation of heat during low-temperature coal oxidation facilitates spontaneous combustion of coal, it is still extremely difficult to simulate such process with relatively fast ReaxFF-MD. Because it is necessary to remove the thermostat for ReaxFF-MD simulation so as to model the heat accumulation when low-temperature coal oxidation occurs. As a result, the temperature increase of the system only relies on the accumulation of heat caused by rare events such as low-temperature coal oxidation. Obviously, enough heat accumulation (e.g., 100 degrees temperature increase) is only possible for large systems which can hardly be studied by molecular dynamics simulations. Our strategy in this manuscript is to simulate the brown coal–oxygen/water systems under different conditions. Hopefully, a series of ReaxFF-MD simulations results can provide us with clues to elucidate spontaneous combustion of brown coal. A more accurate description of spontaneous combustion of coal by ReaxFF-MD might require the application of extreme value theory along with ReaxFF-MD simulations.

## 2. Simulation Models and Methods

**Figure 1:**
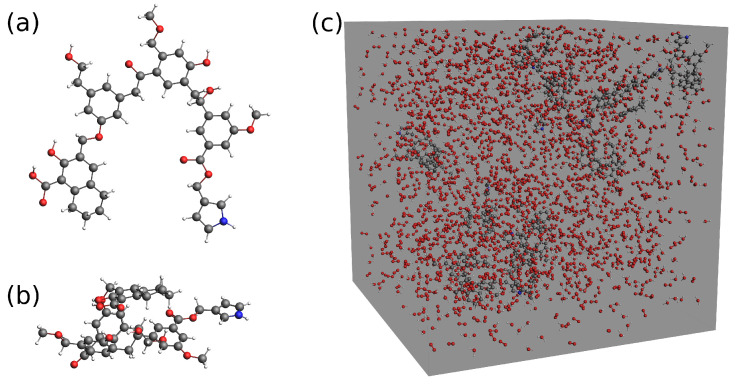
(**a**) The chemical structure of model lignite molecule (C_45_O_12_NH_47_) [41]. The carbon, oxygen, hydrogen, and nitrogen atoms are represented by black, red, white, and blue beads, respectively; (**b**) equilibrated chemical structure of the model lignite molecule; (**c**) an example simulation box of brown coal oxidation (S12 in Table 1).

**Figure 2:**
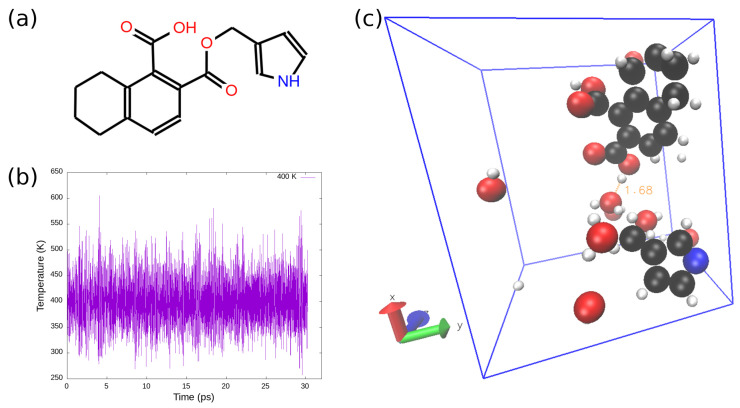
(**a**) The chemical structure of fragment of model lignite molecule (C_17_H_25_O_4_N); (**b**) system temperature as a function of simulation time for NVT ensemble at 400 K; (**c**) snapshot of water-brown coal interaction at 300 K. The carbon, oxygen, hydrogen, and nitrogen atoms are represented by black, red, white, and blue beads, respectively. One example hydrogen bond (1.68 Å) is labeled by an orange dotted line.

In this work, the representative structure of lignite (as shown in Figure 1) developed by Meng et al. [41] was employed to simulate lignite combustion and gasification at various temperatures using ReaxFF molecular dynamics simulations [15,16,17]. Both the chemical structure and the relaxed configuration of the lignite molecular model are given in Figure 1. Ten brown coal molecules were placed in periodic boxes of various sizes (e.g., the simulation system as depicted in Figure 1c). Different numbers of water molecules and/or oxygen molecules were then added into the system to investigate their effects on brown coal oxidation. Detailed information about our simulation systems is presented in Table 1. In addition, the initial configurations of all our ReaxFF-MD simulations were prepared by randomly positioning different numbers of those molecules given in Table 1 in the simulation box, according to their concentrations. Simulation systems were first equilibrated by performing a 10 ps constant-temperature, constant-volume (NVT) simulation at 300 K. In addition, then, the simulation systems were heated to the target temperatures rapidly, followed by another 250 ps NVT simulation except where otherwise stated. Such simulations were performed to investigate the chemical reactions in our simulation systems with a 0.1 femtosecond (fs) timestep, the same as in the equilibration stage. All ReaxFF-MD simulations of brown coal oxidation in this work were performed using the REAX package [13,15,16] of LAMMPS [42] as well as ADF software [43]. Visualization of molecules (Figure 2c) as well as calculation of various coordinate root-mean-squared deviation (RMSD) were carried out by VMD [44].

To compare the simulation results of ReaxFF-MD to that of Car–Parrinello molecular dynamics directly, a small fragment of the original brown coal model molecule (as shown in Figure 2a) was adopted for low-temperature simulations using both ReaxFF-MD and CPMD, as *ab initio* molecular dynamics (CPMD) is much more computationally expensive than ReaxFF-MD. For all low-temperature simulations, one model molecule (C_17_H_25_O_4_N) with eight water molecules were randomly positioned in a 14.4×12.0×12.0 Å${}^{3}$ periodic box, followed by 10 ps NVT ReaxFF-MD equilibration at 300 K. Simulations with this equilibrated initial configuration were performed using both ReaxFF-MD as well as CPMD, as the density of the system was fixed to 0.3565 g/cm${}^{3}$. Car–Parrinello molecular dynamics simulations were carried out using DFT calculation with Perdew–Burke–Ernzerhof (PBE) exchange-correlation functional [45] and Vanderbilt ultrasoft pseudopotentials [46,47,48,49]. As suggested by Vanderbilt et al. [49], a plane-wave cutoff of 40 Ry and a charge-density cutoff of 200 Ry were used for all ab initio molecular dynamics calculations in this manuscript. In addition, the NVT CPMD simulations equipped with a Nosé–Hoover thermostat were run with a time-step of 0.12 fs and a fictitious electronic mass of 400.0 a.u. All the CPMD simulations were performed using the Quantum Espresso package [50,51,52].

## 3. Results and Discussion

### 3.1. Lignite–Water Interaction at Low Temperatures, CPMD vs. ReaxFF-MD

**Figure 3:**
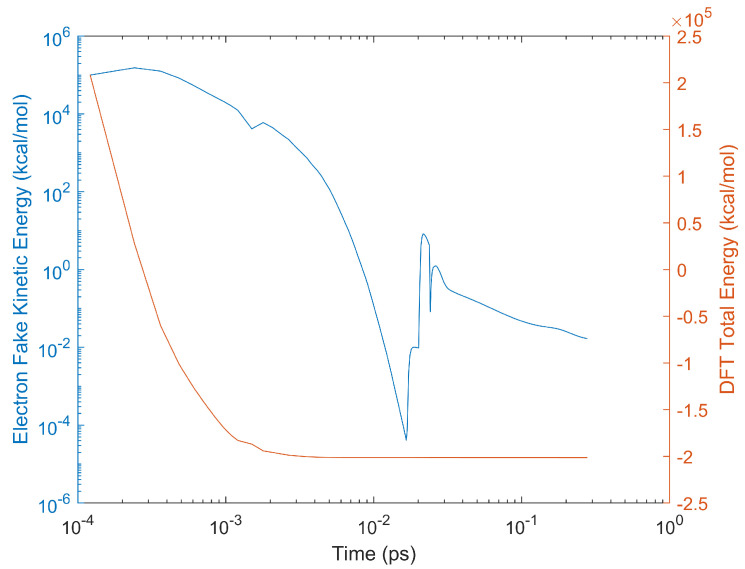
**Cyan curve:** electron fake kinetic energy as a function of relaxation time during relaxation of electronic degrees of freedom as well as structural optimization (ionic and electronic relaxation). **Orange curve:** DFT total energy as a function of relaxation time.

As discussed above, low-rank coal, such as brown coal, contains high moisture content. To examine the adsorptions of water molecules on brown coal (especially on the oxygen-containing polar functional groups), the interactions between brown coal model molecule and water molecules were simulated using both Car–Parrinello molecular dynamics and ReaxFF-MD. For CPMD, both relaxation of electronic degrees of freedom and structural optimization (ionic and electronic relaxation) were carried out before MD runs. As shown in Figure 3, the DFT total energy reaches a plateau after structural optimization. Then, a 3-ps CPMD simulation was performed to equilibrate the system for different temperatures, which was followed by a 30-ps CPMD simulation at the same temperature. For example, the system temperature is plotted against simulation time for NVT ensemble at 400 K, as demonstrated in Figure 2b. For comparison, a 30-ps ReaxFF-MD with the same initial configuration was run at 300 K.

**Figure 4:**
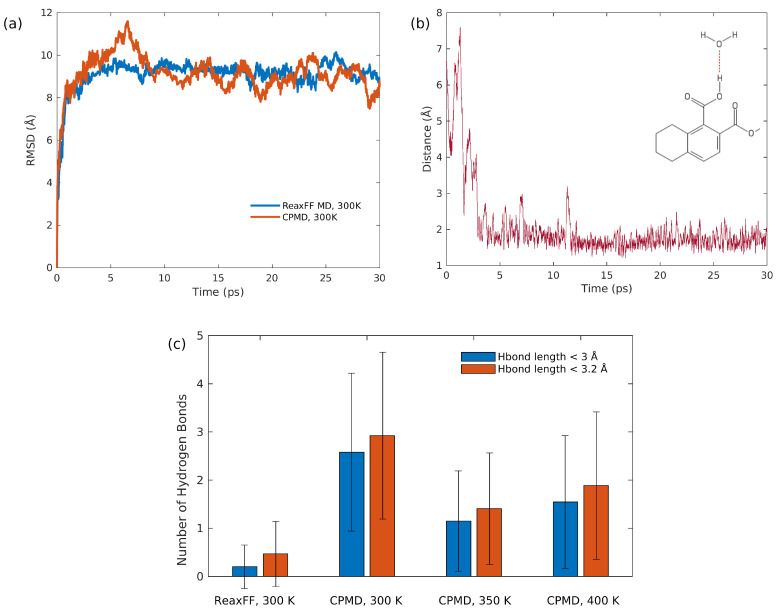
(**a**) Coordinate root-mean-squared deviation (RMSD) of all atoms as a function of time for both ReaxFF-MD and CPMD at 300 K; (**b**) the distance between the hydrogen of the carboxyl group and the oxygen of a water as a function of time; (**c**) average number of hydrogen bonds in the simulation systems obtained by ReaxFF-MD and CPMD. Error bars are standard deviations.

As demonstrated in Figure 4a, the root-mean-squared deviations (RMSD) of all atoms coordinates in simulation box are plotted against a time lag for both ReaxFF-MD as well as CPMD at 300 K. Using the TRAVIS package [53,54], the average diffusion coefficients ($D_{avg}$) of all atoms at 300 K for both ReaxFF-MD and CPMD are obtained which are $1.89\times 10^{-9}$m${}^{2}$/s and $1.53\times 10^{-9}$m${}^{2}$/s, respectively. The good agreement between RMSD/$D_{avg}$ obtained by CPMD and that obtained by ReaxFF-MD simulation shows that the average diffusive behavior of all atoms at low temperature is not affected by dynamical simulation methods. Note that the density of our systems for low temperature calculations was fixed to 0.3565 g/cm${}^{3}$, which is much smaller compared to the density of lignite (∼1.2 g/cm${}^{3}$). Recent ReaxFF molecular dynamics simulations of coal pyrolysis and combustion [6,22,28,35] and CPMD simulations of coal–water interactions [40] mainly focused on coal models with low densities ranging from 0.1∼0.7 g/cm${}^{3}$ [6,22,35]. Therefore, our simulation results can be directly compared to those previous results.

By tracking the trajectories of all atoms during CPMD simulations, a hydrogen bond between the carboxyl group and a water molecule was detected. In addition, the distance between the oxygen of the water molecule and the hydrogen of the carboxyl group is plotted against simulation time as shown in Figure 4b. It can be clearly seen from Figure 4b that, once the hydrogen bond formed, the adsorption of water on carboxyl group remained unchanged for the rest of the simulation. Note that the van der Waals radius of hydrogen atom is 1.06 Å and the van der Waals radius of oxygen atom is 1.42 Å [40,55]. This observation does not agree with the recent simulation results of CPMD with Vanderbilt pseudopotentials obtained by Gao et al. [40], since Gao et al. observed replacement phenomenon of the water during their 30-ps Car–Parrinello molecular dynamics simulations. This disagreement between simulations results might be partly attributed to the fact that Gao et al. [40] chose 25 Ry as the kinetic energy cutoff for wavefunctions instead of 40 Ry, which was suggested by Vanderbilt [48,49], the developer of Vanderbilt pseudopotentials. In addition, Gao et al. [40] have not specified the kinetic energy cutoff for charge density, and the distances between multiple pairs of oxygen and hydrogen exceeded the half-length of the periodic box in their simulations [40], which might be caused by an incorrect analysis.

In order to investigate how the simulation methods and temperature affect hydrogen bond formation in gas phase for our lignite–water system, the average number of hydrogen bonds for both CPMD and ReaxFF-MD are plotted in Figure 4c. In addition, 3.2 Å and 3.0 Å were selected as the statistical distance of hydrogen bonds (H-O···H) to determine whether a hydrogen bond formation took place. As demonstrated in Figure 4c, the average number of hydrogen bonds obtained by ReaxFF-MD is smaller than that obtained by CPMD, which implies that the hydrogen bonds are not accurately described by ReaxFF. In addition, ReaxFF cannot describe the London dispersion (van der Waals (vdW) attraction) accurately [56], which suggests that ReaxFF might not be sufficient to accurately simulate the coal–water interactions at low-temperature. One possible solution to this problem of ReaxFF was proposed by Liu et al. [57], who introduced the low-gradient model to account for the van der Waals attraction. However, their proposed ReaxFF-lg model was based on calculations at room temperature, which might fail at high temperature. Hence, it remains challenging to simulate spontaneous combustion of coal, for an improved unified reactive force field to describe coal oxidation for a wide temperature range is necessary.

### 3.2. Potential Energy vs. Time for ReaxFF-MD Simulations

**Figure 5:**
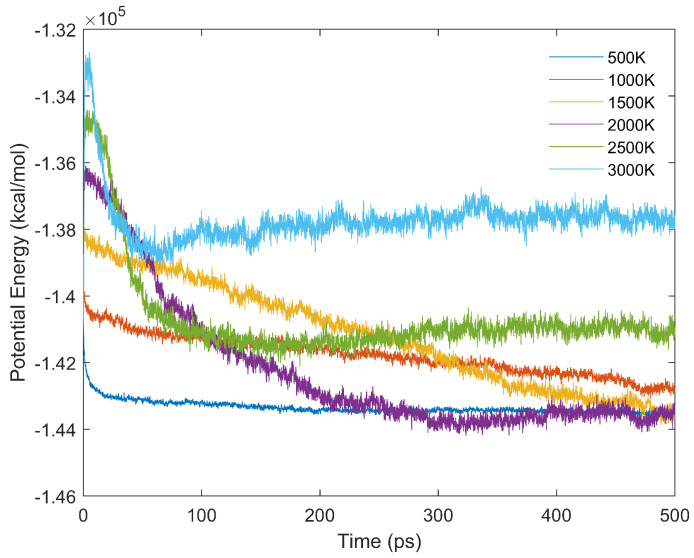
Potential energy of the system (S2 in Table 1) as a function of simulation time at different temperatures.

**Figure 6:**
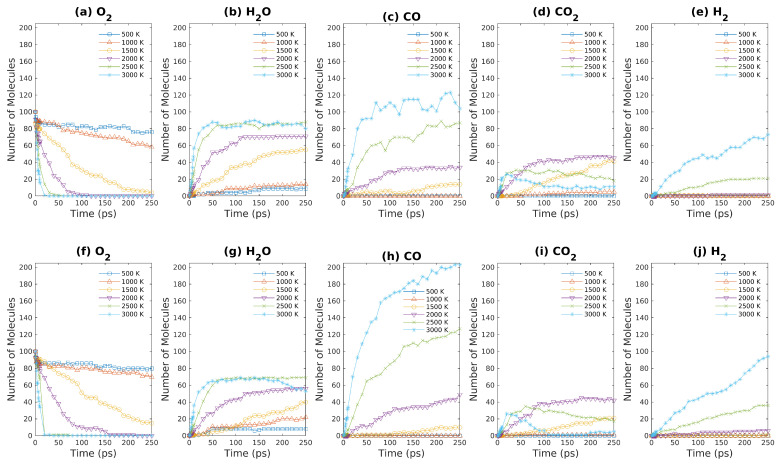
Major products obtained from ReaxFF-MD simulations during oxidation of brown coal at different temperatures. (**a**–**e**): S1 system (Table 1); (**f**–**j**): S2 system (Table 1).

The influence of time step on ReaxFF-MD simulations of hydrocarbon reactive gases has been systematically studied by Jensen et al. [58]. Their results demonstrated that system potential energy converged when the time step is shorter than or equal to 0.1 fs. Therefore, for this manuscript, 0.1 fs was adopted as the time step for ReaxFF-MD simulations. In addition, the potential energy of simulation system (S2 in Table 1) was plotted against simulation time at various temperatures in Figure 5. As shown in Figure 5, the potential energy decreases as time increases, for the combustion/oxidation of brown coal with oxygen is exothermic [22]. In addition, potential energy decreases more rapidly as temperature increases in the early stage, due to the increase in reaction rate. Because the combustion process takes much time [34], a 250-ps ReaxFF-MD simulation is not sufficient to equilibrate the system. For low temperature (500 K), the simulated system reaches equilibrium state rapidly because coal combustion does not occur at this temperature, which is shown by Figure 6. As the rate of coal combustion is enhanced by temperature increase, the potential energy curves keeps going down after 200 ps for 1000∼2000 K systems, while the potential curves above 2000 K reaches a plateau within 200-ps. In other words, systems at higher temperatures achieve the equilibrium state faster.

**Figure 7:**
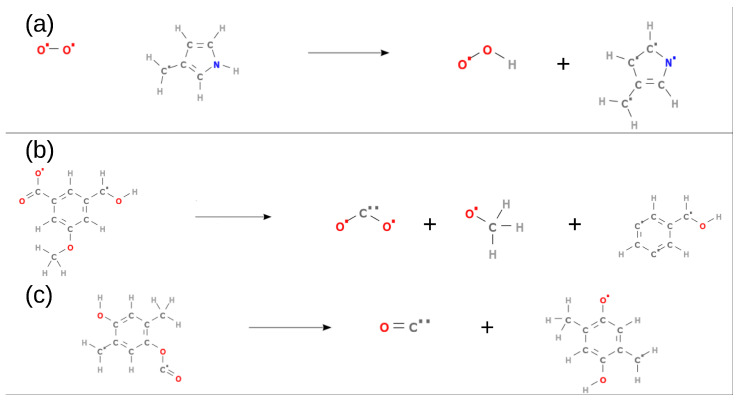
(**a**–**c**) Examples of chemical reactions during ReaxFF-MD simulation at 3000 K.

Reaction mechanisms of brown coal oxidation at different temperatures were analyzed by ADF software [43] with Döntgen’s method [59]. Thousands of chemical species and elementary reactions were observed during our ReaxFF-MD simulations. Several examples of elementary chemical reactions of S2 system in Table 1 at 3000 K are depicted in Figure 7. Similar to the findings of previous studies [6,22,35], our simulation results demonstrated that the brown coal oxidation was initialized by thermal decomposition to produce smaller fragments. Nevertheless, the charge-equilibration (QEq) calculation adopted by ReaxFF-MD has problems that cause unreasonable charge distribution predictions for chemical structures far from equilibrium [56]. As a result, some of our predicted elementary chemical reactions at high temperature might be unreal. Thus, in this manuscript, we focus on CO/CO_2_/H_2_/H_2_O formation which has been extensively investigated using ReaxFF-MD simulations [6,15,16,22,35].

### 3.3. Product Analysis of ReaxFF-MD Simulation Results—Fuel Rich

As described above, a series of ReaxFF-MD simulations of brown coal oxidation were carried out to investigate how the products of brown coal oxidation were affected by temperature as well as coal density. In addition, such simulations were performed for both fuel rich and fuel lean conditions. As demonstrated in Figure 6, the concentrations of major products of S1 and S2 (as shown in Table 1) such as H_2_O, CO, CO_2_, and H_2_ are plotted against simulation time. For S1 & S2 systems, 10 model molecules of brown coal react with 100 oxygen molecules. As shown in Figure 6a,f, both S1 and S2 exhibit sharp decreases in oxygen numbers above 2500 K. On the other hand, the oxygen molecule numbers only decrease slightly when system temperature is less than or equal to 1000 K within 250-ps simulations. Note that, for relatively low temperature (≤1000 K), oxygen molecules are absorbed to brown coal molecules (or fragments) to form some intermediate products, where the final products like CO/CO_2_/H_2_ are negligible (as shown in Figure 6c–e,h–j).

As system temperature increases from 500 K to 3000 K, the number of water molecules increases more and more rapidly over time. For both S1 & S2, the number of water molecules reach a plateau within 50 ps, which seems to suggest that chemical equilibrium is achieved. Nevertheless, as discussed above, the systems cannot be equilibrated within 250-ps ReaxFF-MD simulations even for 3000 K simulations, which can be seen from Figure 6c,e,h,j that CO & H_2_ concentrations keep on increasing during the simulations. As shown in Figure 6g, the curve that shows the relationship between water molecule number and simulation time bends downward after 200 ps simulation time, which indicates that water molecules and carbonaceous structures react to form hydrogen molecules in this stage. For high-temperatures (≥2500 K), the number of carbon dioxide as products increases rapidly in the initial stage and then decreases gradually after 50 ps for fuel rich condition, since CO_2_ is not the preferable product in this condition. The only difference between S1 and S2 is that the density of S2 is only one half of S1 system density. Due to the entropy gain, the number of small inorganic molecules such as CO and H_2_ increases significantly as density decreases, as shown in Figure 6. This fact raises the question whether the reaction pathways observed in previous ReaxFF-MD simulations are applicable to other conditions, especially considering that most previous ReaxFF-MD studies focused on low densities ranging from 0.1–0.7 g/cm${}^{3}$ [6,22,35].

**Figure 8:**
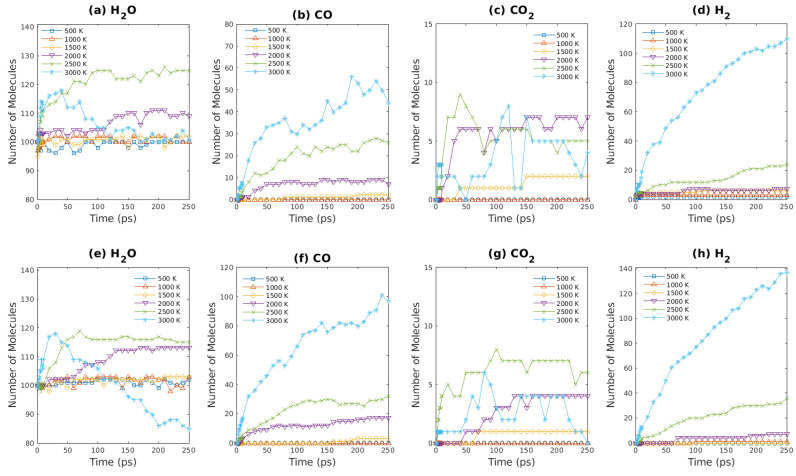
Major products obtained from ReaxFF-MD simulations during oxidation of brown coal at different temperatures. (**a**–**d**): S3 system (Table 1); (**e**–**h**): S4 system (Table 1).

**Figure 9:**
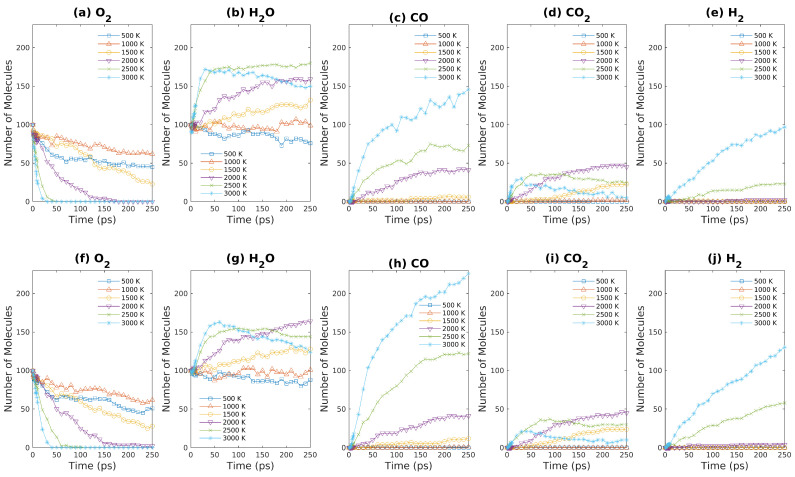
Major products obtained from ReaxFF-MD simulations during oxidation of brown coal at different temperatures. (**a**–**e**): S5 system (Table 1); (**f**–**j**): S6 system (Table 1).

The reaction between brown coal model molecules and water at various temperatures have also been examined by ReaxFF-MD simulation in this work. The numbers of major products of S3 & S4 systems (in Table 1) as functions of simulation time are presented in Figure 8. The number of water molecules almost remains a constant at low temperatures. As temperature increases, the number of water molecules increases over time in the early stage and then enters a plateau region. For the S4 system at 3000 K (in Figure 8e), the number of H_2_O first increases and then decreases over time, which implies the reaction between water molecules and brown coal to produce hydrogen molecules dominates after 50 ps for the fuel rich condition. Due to the lacking of oxygen molecules, CO production is favored compared to CO_2_ production, especially at high temperatures. Hence, although the numbers of water molecules and CO_2_ molecules vary insignificantly, the numbers of CO and H_2_ increase rapidly above 2500 K. Again, due to the entropy gain as a result of increase of simulation box size, the numbers of CO and H_2_ molecules increase more quickly as system density is reduced by half. As demonstrated in Figure 9, the concentrations of major products of S5 & S6 systems (in Table 1) at different temperatures obtained by ReaxFF-MD simulations are plotted against simulation time. As oxygen and water molecules coexist in the S5 & S6 systems for the initial configuration, the number of oxygen molecules decrease faster than that of S1 & S2 systems, which suggests that the interactions among brown coal, water, and oxygen might be cooperative. Figure 9b,g demonstrate that the number of water molecules increases rapidly during the first 50 ps simulation and then decreases gradually for high temperatures, which indicates that water molecules react with lignite to produce hydrogen molecules in this condition. Due to insufficient oxygen present in this combustion reaction, incomplete conversion takes place for this fuel rich condition where a large amount of CO molecules have formed as shown in Figure 9c,h. Note that our simulation results (Figure 9d,i) indicate that CO_2_ production is favored at lower temperature (2000 K), whereas CO production is favored at higher temperature (3000 K), which agrees with previous research [22]. In addition, hydrogen molecules only form at high temperatures (2500∼3000 K). When the system density is reduced by half, the amount of gaseous products like CO, H_2_ increase significantly, which again confirms the important effects of density on brown coal oxidation.

**Figure 10:**
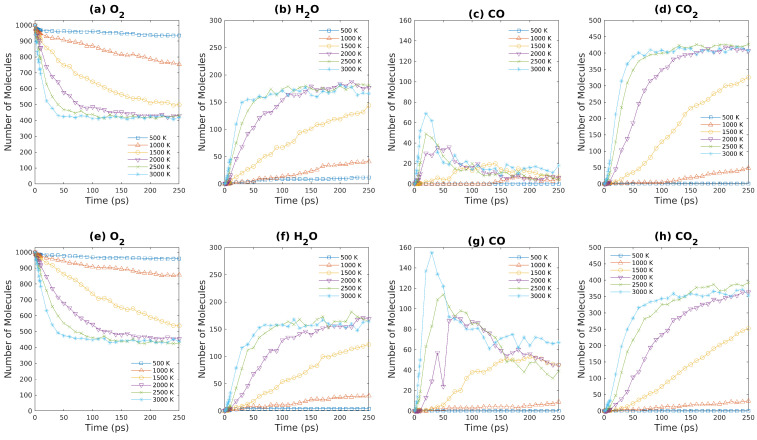
Major products obtained from ReaxFF-MD simulations during oxidation of brown coal at various temperatures. (**a**–**d**): S7 system (Table 1); (**e**–**h**): S8 system (Table 1).

For comparison, the reactions between 10 brown coal model molecules and 1000 water molecules (S9 & S10) were also investigated using ReaxFF-MD simulations. In addition, the amounts of gaseous products as functions of simulation time are plotted in Figure 10. Since there are 1000 water molecules in S9 & S10 systems, the number densities of brown coal molecules in S9 & S10 systems are less than that in S3 & S4 systems. Changes in numbers of water molecules in the S9 & S10 systems are relatively small except S10 at 3000 K. For S10 at 3000 K, the number of water molecules decreases significantly over time, which indicates that the reaction between water and brown coal to produce hydrogen molecules is sped up appreciably at 3000 K. By comparing Figure 8b to Figure 10b (or, Figure 8d vs. Figure 10d, Figure 8f vs. Figure 10f, Figure 8f vs. Figure 10f), we can conclude that the production rates of CO/H_2_ molecules in S9 & S10 systems are similar to that in S3 & S4 systems. For this fuel rich condition, the CO_2_ production can be neglected compared to CO production. Moreover, by reducing the system density by half, the number of hydrogen molecules increase from ∼140 to ∼200 within the 250 ps ReaxFF-MD simulations.

### 3.4. Product Analysis of ReaxFF-MD Simulation Results—Fuel Lean

**Figure 11:**
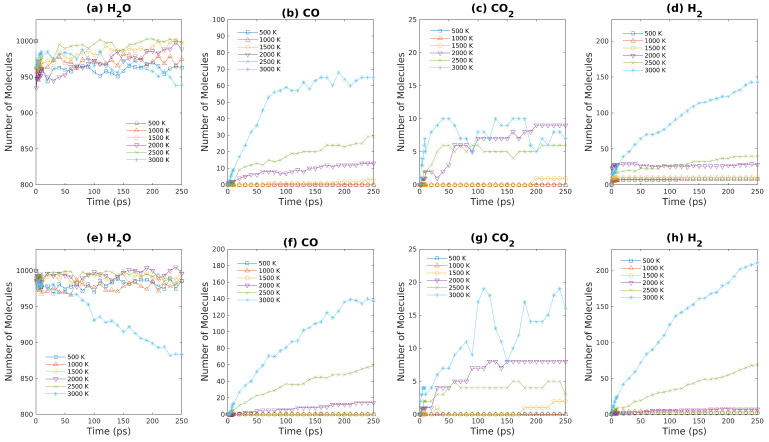
Major products obtained from ReaxFF-MD simulations during oxidation of brown coal at various temperatures. (**a**–**d**): S9 system (Table 1); (**e**–**h**): S10 system (Table 1).

**Figure 12:**
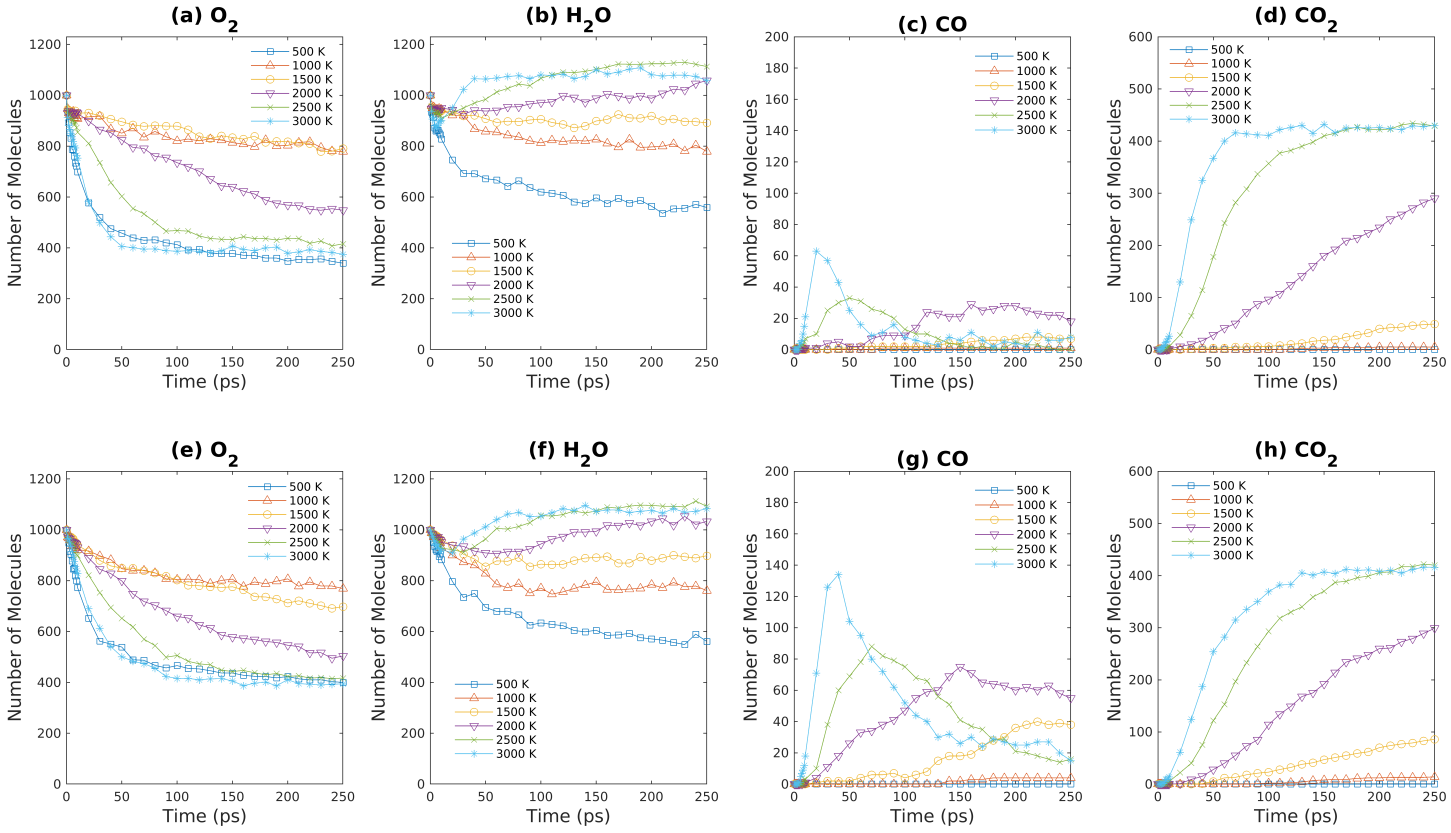
Major products obtained from ReaxFF-MD simulations during oxidation of brown coal at various temperatures. (**a**–**d**): S11 system (Table 1); (**e**–**h**): S12 system (Table 1).

The amounts of gaseous products as functions of simulation time are plotted in Figure 11 and Figure 12 for fuel lean conditions. Due to the larger number of O_2_ molecules present in systems, the rate of product generation is greater in comparison to fuel rich condition, which is evident by Figure 11d,h. Such observation in our simulations also agrees with previous experimental results [60,61] and simulation results [22]. The excess of oxygen molecules in S7 & S8 allows the complete combustion of brown coal to take place. As shown in Figure 11d,h, the number of CO_2_ increases rapidly at the beginning and then enters a plateau region within 250 ps for high temperatures (2000∼3000 K), since most carbon atoms are changed into carbon dioxide. Note that the amount of CO increases at 3000 K initially and then decreases rapidly as CO is consumed by combustion for the fuel lean condition. In addition, since complete combustion of brown coal occurs for fuel lean conditions, the entropy gain due to the density change has a negligible effect on brown coal oxidation in this condition.

As shown in Figure 12, the variation of gaseous products with time is plotted. The number of oxygen molecules in S11/S12 decreases faster than that in S7/S8 as water molecules facilitate the reactions between oxygen molecules and brown coal. The CO/CO_2_ production in S11 & S12 is similar to that in S7 & S8 because the oxygen molecules that are abundant in systems that complete combustions take place. However, as demonstrated in Figure 12c,g, the amount of carbon oxide increases as density is reduced by half. This increase in number of CO molecules can be attributed to the fact that CO is more stable at high temperature (3000 K), which agrees with previous results [22]. As discussed above, because brown coal combustion for fuel lean conditions is almost complete, the entropy change due to density difference has a negligible effect on final gaseous products formations.

## 4. Conclusions

In this manuscript, using a combination of ab initio molecular dynamics simulation (CPMD) and ReaxFF reactive molecular dynamics simulation, the interactions between water molecules and brown coal molecular model were investigated. Our CPMD simulation results demonstrate that hydrogen bonds formed between water and oxygen-containing polar functional groups contained in lignite, especially carboxyl, dominate the interaction between water and coal. Our simulation results not only confirm the results of previous quantum chemistry calculations [8,9], but also provide extra statistical information of the brown coal–water systems as system entropy is absent in those quantum chemistry calculations. The comparison between our CPMD simulations results and our ReaxFF-MD simulation results suggests that the ReaxFF reactive force field is not accurate enough to describe the diffusive behaviors of water molecules on the brown coal molecular model. Although the low-gradient method might correct the ReaxFF reactive force field at room temperature, it is still difficult to simulate temperature dependent diffusion of water on lignite, since the low-gradient method is based on the system configuration at a fixed low temperature [57].

Our ReaxFF-MD simulation results reveal the mechanisms in which the brown coal molecular model is oxidized for a wide temperature range. By varying the system density, it has been observed that the generation of gaseous products is sped up by density decrease due to the entropy gain. Hence, our simulation results as well as previous ReaxFF-MD simulations of coal combustion might not be able to describe the spontaneous combustion of coal piles as ReaxFF-MD simulations mainly focus on low density systems. Moreover, Zheng et al. [62] demonstrated that many reaction pathways can only be detected using ReaxFF-MD with a large molecular model of coal. Therefore, in order to study the low temperature oxidation of brown coal to elucidate the reaction mechanisms of coal spontaneous combustion, a more condensed phase of large brown coal molecular model with air is required.

The combination of CPMD and ReaxFF-MD simulations allows us to partially overcome the limitations of conventional force field, as we seek to study systematically the correlated water transportation and brown coal oxidation at various temperatures. However, a modified version of ReaxFF reactive force field which can accurately describe the temperature dependent diffusion of gases (e.g., water) on lignite as well as a large brown coal molecular model with appropriate density are required to investigate the complicated reactions during coal spontaneous combustion/gasification.

## Figures and Tables

**Table 1:** Parameters of reaction systems.

System	Number of	Number of	Number of	Density	Dimension
	C_45_O_12_NH_47_	H_2_O	O_2_	(g/cm${}^{3}$)	(Å)
S1	10	0	100	0.7130	29.603 × 29.603 × 29.603
S2	10	0	100	0.3565	37.298 × 37.298 × 37.298
S3	10	100	0	0.7130	28.308 × 28.308 × 28.308
S4	10	100	0	0.3565	35.666 × 35.666 × 35.666
S5	10	100	100	0.7130	31.120 × 31.120 × 31.120
S6	10	100	100	0.3565	39.208 × 39.208 × 39.208
S7	10	0	1000	0.7130	45.308 × 45.308 × 45.308
S8	10	0	1000	0.3565	57.085 × 57.085 × 57.085
S9	10	1000	0	0.7130	39.245 × 39.245 × 39.245
S10	10	1000	0	0.3565	49.445 × 49.445 × 49.445
S11	10	1000	1000	0.7130	51.296 × 51.296 × 51.296
S12	10	1000	1000	0.3565	64.631 × 64.631 × 64.631

## Data Availability

The data presented in this study are openly available in Mendeley Data at http://dx.doi.org/10.17632/k3n53ts7rr.1 (accessed on 27 December 2021).

## References

[1] BP BP Statistical Review of World Energy. https://www.bp.com/content/dam/bp/business-sites/en/global/corporate/pdfs/energy-economics/statistical-review/bp-stats-review-2020-full-report.pdf.

[2] Yu J., Tahmasebi A., Han Y., Yin F., Li X. (2013). A review on water in low rank coals: The existence, interaction with coal structureand effects on coal utilization. Fuel Process. Technol..

[3] Wu J., Liu J., Yuan S., Wang Z., Zhou J., Cen K. (2016). Theoretical Investigation of noncovalent interactions between low-rank coal and water. Energy Fuels.

[4] Xu C., Xu G., Zhao S., Zhou L., Yang Y., Zhang D. (2015). An improved configuration of lignite pre-drying using a supplementary steam cycle in a lignite fired supercritical power plant. Appl. Energy.

[5] Arisoy A., Akgün F. (1994). Modelling of spontaneous combustion of coal with moisture content included. Fuel.

[6] Zhou Z., Guo L., Chen L., Shan S., Wang Z. (2018). Study of pyrolysis of brown coal and gasification of coal–water slurry using the ReaxFF reactive force field. Int. J. Energy Res..

[7] Wang Y., Zhou J., Bai L., Chen Y., Zhang S., Lin X. (2014). Impacts of inherent O-containing functional groups on the surface properties of Shengli lignite. Energy Fuels.

[8] Gao Z., Ding Y., Yang W., Han W. (2017). DFT study of water adsorption on lignite molecule surface. J. Mol. Model..

[9] Xiao J., Zhao Y.P., Fan X., Cao J.P., Kang G.J., Zhao W., Wei X.Y. (2017). Hydrogen bonding interactions between the organic oxygen/nitrogen monomers of lignite and water molecules: A DFT and AIM study. Fuel Process. Technol..

[10] Suzuki A., Yamamoto T., Aoki H., Miura T. (2002). Percolation model for simulation of coal combustion process. Proc. Combust. Inst..

[11] Guo X., Liu Z., Xiao Y., Xu X., Xue X., Liu Q. (2018). The Boltzmann-Monte-Carlo-Percolation (BMCP) model on pyrolysis of coal: The volatiles’ reactions. Fuel.

[12] Li X., Grace J.R., Watkinson A.P., Lim C.J., Ergüdenler A. (2001). Equilibrium modeling of gasification: A free energy minimization approach and its application to a circulating fluidized bed coal gasifier. Fuel.

[13] Van Duin A.C.T., Dasgupta S., Lorant F. (2001). ReaxFF: A Reactive Force Field for Hydrocarbons. J. Phys. Chem. A.

[14] Senftle T.P., Hong S., Islam M.M., Kylasa S.B., Zheng Y., Shin Y.K., Junkermeier C., Engel-Herbert R., Janik M.J., Aktulga H.M. (2016). The ReaxFF reactive force-field: Development, applications and future directions. NPJ Comput. Mater..

[15] Strachan A., van Duin A.C.T., Chakraborty D., Dasgupta S., Goddard W.A. (2003). Shock Waves in High-Energy Materials: The Initial Chemical Events in Nitramine RDX. Phys. Rev. Lett..

[16] Strachan A., Kober E.M., van Duin A.C.T., Oxgaard J., Goddard W.A. (2005). Thermal decomposition of RDX from reactive molecular dynamics. J. Chem. Phys..

[17] Zhang L., van Duin A.C.T., Zybin S.V., Goddard W.A. (2009). Thermal decomposition of hydrazines from reactive dynamics using the ReaxFF reactive force field. J. Phys. Chem. B.

[18] Chenoweth K., van Duin A.C.T., Goddard W.A. (2008). ReaxFF reactive force field for molecular dynamics simulations of hydrocarbon oxidation. J. Phys. Chem. A.

[19] Verma A., Parashar A. (2018). Reactive force field based atomistic simulations to study fracture toughness of bicrystalline graphene functionalised with oxide groups. Diam. Relat. Mater..

[20] Goverapet Srinivasan S., van Duin A.C.T., Ganesh P. (2015). Development of a ReaxFF potential for carbon condensed phases and its application to the thermal fragmentation of a large fullerene. J. Phys. Chem. A.

[21] Zheng M., Li X., Guo L. (2013). Algorithms of GPU-enabled reactive force field (ReaxFF) molecular dynamics. J. Mol. Graph. Model..

[22] Bhoi S., Banerjee T., Mohanty K. (2014). Molecular dynamic simulation of spontaneous combustion and pyrolysis of brown coal using ReaxFF. Fuel.

[23] Mathews J.P., van Duin A.C.T., Chaffee A.L. (2011). The utility of coal molecular models. Fuel Process. Technol..

[24] Mathews J.P., Chaffee A.L. (2012). The molecular representations of coal—A review. Fuel.

[25] Salmon E., van Duin A.C.T., Lorant F., Marquaire P.M., Goddard W.A. (2009). Early maturation processes in coal. Part 2: Reactive dynamics simulations using the ReaxFF reactive force field on Morwell Brown coal structures. Org. Geochem..

[26] Zheng M., Li X., Liu J., Guo L. (2013). Initial chemical reaction simulation of coal pyrolysis via ReaxFF molecular dynamics. Energy Fuels.

[27] Zhan J.H., Wu R., Liu X., Gao S., Xu G. (2014). Preliminary understanding of initial reaction process for subbituminous coal pyrolysis with molecular dynamics simulation. Fuel.

[28] Hong D., Guo X. (2017). Molecular dynamics simulations of Zhundong coal pyrolysis using reactive force field. Fuel..

[29] Zheng M., Li X., Guo L. (2018). Investigation of N behavior during coal pyrolysis and oxidation using ReaxFF molecular dynamics. Fuel.

[30] Hong D., Liu L., Zhang S., Guo X. (2018). Effect of cooling rate on the reaction of volatiles from low-rank coal pyrolysis: Molecular dynamics simulations using ReaxFF. Fuel Process. Technol..

[31] Zhang G., Li J., Zhang M., Sun S., Luo Y. (2018). Multistep pyrolysis behavior of core-shell type hyperbranched azide copolymer: Kinetics and reaction mechanism via experiment and simulation. Fuel.

[32] Zhang T., Li X., Guo L. (2017). Initial reactivity of linkages and monomer rings in lignin pyrolysis revealed by ReaxFF molecular dynamics. Langmuir.

[33] Zhong Q., Mao Q., Xiao J., van Duin A., Mathews J.P. (2018). Sulfur removal from petroleum coke during high-temperature pyrolysis. Analysis from TG-MS data and ReaxFF simulations. J. Anal. Appl. Pyrolysis.

[34] Castro-Marcano F., Kamat A.M., Russo M.F., van Duin A.C.T., Mathews J.P. (2012). Combustion of an Illinois No. 6 coal char simulated using an atomistic char representation and the ReaxFF reactive force field. Combust. Flame..

[35] Jin H., Xu B., Li H., Ku X., Fan J. (2018). Numerical investigation of coal gasification in supercritical water with the ReaxFF molecular dynamics method. Int. J. Hydrog. Energy.

[36] Xie L., Shao Y., Zhong W., Ben H., Li K. (2019). Molecular dynamic simulation on the oxidation process of coal tar pitch. Fuel.

[37] You J., Tian L., Zhang C., Yao H., Dou W., Fan B., Hu S. (2016). Adsorption behavior of carbon dioxide and methane in bituminous coal: A molecular simulation study. Chin. J. Chem. Eng..

[38] Dang Y., Zhao L., Lu X., Xu J., Sang P., Guo S., Zhu H., Guo W. (2017). Molecular simulation of CO_2_/CH_4_ adsorption in brown coal: Effect of oxygen-, nitrogen-, and sulfur-containing functional groups. Appl. Surf. Sci..

[39] Wu S., Jin Z., Deng C. (2019). Molecular simulation of coal-fired plant flue gas competitive adsorption and diffusion on coal. Fuel.

[40] Gao Z., Ma C., Lv G., Li A., Li X., Liu X., Yang W. (2019). Car–Parrinello molecular dynamics study on the interaction between lignite and water molecules. Fuel..

[41] Meng X., Gao M., Chu R., Miao Z., Wu G., Bai L., Liu P., Yan Y., Zhang P. (2017). Construction of a macromolecular structural model of Chinese lignite and analysis of its low-temperature oxidation behavior. Chin. J. Chem. Eng..

[42] Plimpton S. (1995). Fast Parallel Algorithms for Short-Range Molecular Dynamics. J. Comp. Phys..

[43] te Velde G., Bickelhaupt F.M., Baerends E.J., Fonseca Guerra C., van Gisbergen S.J.A., Snijders J.G., Ziegler T. (2001). Chemistry with ADF. J. Comput. Chem..

[44] Humphrey W., Dalke A., Schulten K. (1996). VMD–Visual Molecular Dynamics. J. Molec. Graphics.

[45] Perdew J.P., Burke K., Ernzerhof M. (1996). Generalized gradient approximation made simple. Phys. Rev. Lett..

[46] Vanderbilt D. (1990). Soft self-consistent pseudopotentials in a generalized eigenvalue formalism. Phys. Rev. B.

[47] Laasonen K., Car R., Lee C., Vanderbilt D. (1991). implementation of ultrasoft pseudopotentials in *ab initio* molecular dynamics. Phys. Rev. B.

[48] Laasonen K., Pasquarello A., Car R., Lee C., Vanderbilt D. (1993). Car–Parrinello molecular dynamics with Vanderbilt ultrasoft pseudopotentials. Phys. Rev. B.

[49] Garrity K.F., Bennett J.W., Rabe K.M., Vanderbilt D. (2014). Pseudopotentials for high-throughput DFT calculations. Comput. Mater. Sci..

[50] Giannozzi P., Baroni S., Bonini N., Calandra M., Car R., Cavazzoni C., Ceresoli D., Chiarotti G.L., Cococcioni M., Dabo I. (2009). QUANTUM ESPRESSO: A modular and open-source software project for quantum simulations of materials. J. Phys. Condens. Matter..

[51] Giannozzi P., Baseggio O., Bonfá P., Brunato D., Car R., Carnimeo I., Cavazzoni C., de Gironcoli S., Delugas P., Ruffino F.F. (2020). Quantum ESPRESSO toward the exascale. J. Chem. Phys..

[52] Car R., Parrinello M. (1985). Unified Approach for Molecular Dynamics and Density-Functional Theory. Phys. Rev. Lett..

[53] Brehm M., Kirchner B. (2011). TRAVIS—A free analyzer and visualizer for Monte Carlo and molecular dynamics trajectories. J. Chem. Inf. Model..

[54] Brehm M., Thomas M., Gehrke S., Kirchner B. (2020). TRAVIS—A free analyzer for trajectories from molecular simulation. J. Chem. Phys..

[55] Bondi A. (1964). Van der Waals volumes and radii. J. Phys. Chem..

[56] Han Y., Jiang D., Zhang J., Li W., Gan Z., Gu J. (2016). Development, applications and challenges of ReaxFF reactive force field in molecular simulations. Front. Chem. Sci. Eng..

[57] Liu L., Liu Y., Zybin S.V., Sun H., Goddard W.A. (2011). ReaxFF-lg: Correction of the ReaxFF reactive force field for London dispersion, with applications to the equations of state for energetic materials. J. Phys. Chem. A.

[58] Jensen B.D., Bandyopadhyay A., Wise K.E., Odegard G.M. (2012). Parametric study of ReaxFF simulation parameters for molecular dynamics modeling of reactive carbon gases. J. Chem. Theory Comput..

[59] Döntgen M., Przybylski-Freund M.D., Kröger L.C., Kopp W.A., Ismail A.E., Leonhard K. (2015). Automated Discovery of Reaction Pathways, Rate Constants, and Transition States Using Reactive Molecular Dynamics Simulations. J. Chem. Theory Comput..

[60] Niksa S., Liu G.S., Hurt R.H. (2003). Coal conversion sub-models for design applications at elevated pressures. Part I. devolatilization and char oxidation. Prog. Energy Combust. Sci..

[61] Liu G.S., Niksa S. (2004). Coal conversion sub-models for design applications at elevated pressures. Part II. Char gasification. Prog. Energy Combust. Sci..

[62] Zheng M., Li X., Nie F., Li G. (2017). Investigation of model scale effects on coal pyrolysis using ReaxFF MD simulation. Mol. Simul..

